# Ectopic Expression of Homeobox Gene NKX2-1 in Diffuse Large B-Cell Lymphoma Is Mediated by Aberrant Chromatin Modifications

**DOI:** 10.1371/journal.pone.0061447

**Published:** 2013-04-29

**Authors:** Stefan Nagel, Stefan Ehrentraut, Jürgen Tomasch, Hilmar Quentmeier, Corinna Meyer, Maren Kaufmann, Hans G. Drexler, Roderick A. F. MacLeod

**Affiliations:** 1 Department of Human and Animal Cell Lines, Leibniz-Institute DSMZ–German Collection of Microorganisms and Cell Cultures, Braunschweig, Germany; 2 Microbial Communication, Helmholtz Centre for Infection Research, Braunschweig, Germany; INRS, Canada

## Abstract

Homeobox genes encode transcription factors ubiquitously involved in basic developmental processes, deregulation of which promotes cell transformation in multiple cancers including hematopoietic malignancies. In particular, NKL-family homeobox genes TLX1, TLX3 and NKX2-5 are ectopically activated by chromosomal rearrangements in T-cell neoplasias. Here, using transcriptional microarray profiling and RQ-PCR we identified ectopic expression of NKL-family member NKX2-1, in a diffuse large B-cell lymphoma (DLBCL) cell line SU-DHL-5. Moreover, in silico analysis demonstrated NKX2-1 overexpression in 5% of examined DLBCL patient samples. NKX2-1 is physiologically expressed in lung and thyroid tissues where it regulates differentiation. Chromosomal and genomic analyses excluded rearrangements at the NKX2-1 locus in SU-DHL-5, implying alternative activation. Comparative expression profiling implicated several candidate genes in NKX2-1 regulation, variously encoding transcription factors, chromatin modifiers and signaling components. Accordingly, siRNA-mediated knockdown and overexpression studies confirmed involvement of transcription factor HEY1, histone methyltransferase MLL and ubiquitinated histone H2B in NKX2-1 deregulation. Chromosomal aberrations targeting MLL at 11q23 and the histone gene cluster HIST1 at 6p22 which we observed in SU-DHL-5 may, therefore, represent fundamental mutations mediating an aberrant chromatin structure at NKX2-1. Taken together, we identified ectopic expression of NKX2-1 in DLBCL cells, representing the central player in an oncogenic regulative network compromising B-cell differentiation. Thus, our data extend the paradigm of NKL homeobox gene deregulation in lymphoid malignancies.

## Introduction

Lymphocytes originate from hematopoietic stem cells located in the bone marrow. While T-cells complete their development in the thymus, B-cells differentiate in various lymphoid tissues. Lymphoid malignancies emerge in the bone marrow or in secondary hematopoietic organs, acquiring both general and subtype specific mutations including chromosomal rearrangements. Accordingly, subtypes of the diffuse large B-cell lymphoma (DLBCL) differ in mutations and gene activities [1]. The sub-classification of this type of hematopoietic cancer represents a milestone in oncological research and has extensive implications for diagnosis and therapy. Two major subtypes, namely germinal center-derived B-cell and activated B-cell, are distinguished within the DLBCL entity [2]. It is believed that additional stratification should contribute to improved and better targeted therapies. Therefore, identification of novel genes or gene networks with diagnostic or therapeutic potential is of clinical interest.

Deregulated genes in leukemia/lymphoma comprise activated transcription factors (TFs) and signaling components which are either physiologically expressed in early stages of hematopoietic development or ectopically induced. Notable examples include TFs of the basic helix-loop-helix (bHLH) family or constituents of the NOTCH-signaling pathway [3]. The NOTCH gene itself may be activated by rare chromosomal translocations in T-cell acute lymphoblastic leukemia/lymphoma (T-ALL) and by mutations affecting both T-ALL and B-cell malignancies. Targets of NOTCH-signaling comprise MYC and bHLH genes HES1 and HEY1 which may represent key oncogenes in malignant transformation [4].

Homeobox genes encode transcription factors frequently deregulated in cancers, including leukemia/lymphoma, impacting developmental processes during embryogenesis. According to their conserved homeobox sequences, this group of TFs has been classified into several subfamilies [5]. NKL family members regulate mesodermal differentiation and organogenesis [6], including NKX2-1 which regulates development of lung and thyroid, together with NKX2-5 and NKX3-1 which regulate that of the heart and prostate, respectively [7]–[10]. NKL-family members are involved in T-ALL [11], where activation usually follows chromosomal juxtaposition to potent transcriptional enhancers cognate to T-cell receptor genes at 7p14, 7q35 and 14q11, or the TF encoding gene BCL11B at 14q32 [12]. Exceptional, NKL family member NKX3-1 is ectopically expressed in T-ALL cells by the activating TFs TAL1, LYL1 and MSX2 rather than cytogenetically [13], [14].

On the other hand the clustered HOX genes are usually activated by formation of aberrant chromatin structures in leukemia/lymphoma, although chromosomal aberrations are described in T-ALL [15]. Specific covalent modifications of core histones mediated by mutated MLL represent the most frequent mechanism of chromatin deregulation activating this homeobox gene group, including HOXA5 and HOXA10 [16]. MLL encodes a histone H3 methyltransferase and is associated with many cofactors in a ternary complex. Moreover, several genes encoding these cofactors are involved in fusion configurations with the MLL gene [17].

Here, we investigate aberrant expression of NKL homeobox gene NKX2-1 in B-cell lymphoma cell line SU-DHL-5. Our data expand the oncogenic role of NKL homeobox genes within the lymphoid system encompassing the B-cell lineage. We demonstrate mechanisms of NKX2-1 activation in addition to examining its downstream effects which include deregulation of cell differentiation in DLBCL.

## Materials and Methods

### Cell lines and treatments

Authenticated mycoplasma-free cell lines were drawn from the DSMZ Cell Lines Bank (Braunschweig, Germany) and cultivated as described previously [18]. Expression constructs and small interfering (si)RNAs were transfected into the cells by electroporation using the EPI-2500 impulse generator (Fischer, Heidelberg, Germany) at 350 V for 10 ms and subsequently incubated for 16 h. Expression constructs for HEY1 and HES1 were obtained from Origene (Wiesbaden, Germany). SiRNA oligonucleotides and AllStars negative Control siRNA (termed here siCTR) were obtained from Qiagen (Hilden, Germany). Cell stimulations were performed by treatment with recombinant human Bone morphogenetic protein (BMP) 4, Interleukin (IL)4, IL10, Transforming growth factor beta (TGFb), Tumor necrosis factor alpha (TNFa), or WNT5B for 16 h at concentrations of 20 ng/ml (R & D Systems, Wiesbaden, Germany). Treatments with γ-secretase inhibitor N-[N-(3,5-Difluorophenacetyl)-L-alanyl]-S-phenylglycine t-butyl ester (DAPT) (Sigma, Taufkirchen, Germany) were performed at concentrations of 1 µM for 16 h, with cAMP-derivate (8-4-Chlorophenylthio-adenosine 3′,5′-cyclic monophosphate, Sigma) and cGMP-derivate (8-bromoguanosine 3′,5′-cyclic monophosphate, Sigma) at concentrations of 100 µM, with Sildenafil (Tocris Bioscience, Bristol, UK) at concentrations of 100 µM and with the NFkB-inhibitor (Calbiochem, Darmstadt, Germany) as indicated in the text. Treatments with anti-TGF beta-1 (clone 9016.2, Thermo Scientific, Germany) were performed with 1 µg/ml for 16 h. DZNep was kindly provided by Dr. Marquez (NIH, Frederick, MD, USA) and applied as described recently [19].

### Expression profiling

For quantification of gene expression via profiling, we used data obtained by gene chips HG U133 Plus 2.0 from Affymetrix (High Wycombe, UK). The datasets were generated at the University of Würzburg and generously provided by Prof. Andreas Rosenwald (Institute of Pathology, University of Würzburg, Germany) or obtained from the National Center for Biotechnology Information (NCBI) Gene Expression Omnibus (GEO) (www.ncbi.nlm.nih.gov/gds) or from the National Cancer Institute (NCI). The NCI microarray data for SU-DHL-5 are available through the accession numbers SU-DHL-5_SS392729_HG-U133_Plus_2_HCHP-201545_.CEL, SU-DHL-5 _SS392730_HG-U133_Plus_2_HCHP-201546_.CEL, and SU-DHL-5_SS392731_HG-U133_Plus_2_HCHP-201547_.CEL which were combined in this study. Analyses of expression data were performed using Microsoft Excel and online programs. For creation of heat maps we used CLUSTER version 2.11 and TREEVIEW version 1.60 (http://rana.lbl.gov/EisenSoftware.htm). Expression data of 203 DLBCL patient samples were obtained from the NCBI GEO database (accession number GSE11318) as published recently [20]. Statistical analyses of NKX2-1 (dataset 211024_s_at) expression were performed using R-software.

### Genomic array analysis

Genome-wide copy number analysis was performed using the Affymetrix Genotyping Console GTC Software version 4.0 (Affymetrix) and visualized by the Affymetrix GTC-Browser program. The 500K-array dataset for SU-DHL-5 was obtained from the National Cancer Institute (Bethesda, MD, USA), GSK Cancer Cell Line Genomic Profiling Data (https://cabig.nci.nih.gov/community/caArray_GSKdata/).

### Chromosomal analyses

Fluorescent in-situ hybridization (FISH) and spectral karyotyping (SKY) analyses were performed as described previously [21], [22]. RP11-BAC-clones were obtained from BacPac Resources, Childreńs Hospital Oakland Research Institute (CA, USA), prepared using the Big BAC DNA Kit (Princeton Separations, Adelphia, NJ, USA) and directly labelled by nick translation with dUTP-fluors (Dyomics, Jena, Germany). For analysis of the NKX2-1 locus BACs RP11-676A19, RP11-945C4, and RP11-74D5 were labelled with Dy-590, Dy-547 and Dy-495, respectively. For analysis of the HEY1 locus we used RP11-300M12 (Dy-590), RP11-89I14 (Dy-547) and RP11-24P11 (Dy-495), for that of MLL we used RP11-347D24 (Dy-547), RP11-91A14 (Dy-590) and RP11-770K18 (Dy-495), and for HIST1 RP11-958P15 (Dy-495), RP11-846O7 (Dy-547) and RP11-6N6 (Dy-590). Fluorescence images were captured and analyzed with an Axio-Imager microscope (Zeiss, Göttingen, Germany) configured to a dual Spectral Imaging FISH and SKY system (Applied Spectral Imaging, Neckarhausen,Germany).

### Polymerase chain-reaction (PCR) analyses

Total RNA from cell lines was extracted using TRIzol reagent (Invitrogen, Karlsruhe, Germany). Total human RNA isolated from peripheral blood mononuclear cells (PBMC), bone marrow (BM), lymph nodes (LN), thymus, lung and thyroid was obtained from Clontech (Saint-Germain-en-Laye, France), and RNA isolated from CD3-positive T-cells and CD19-positive B-cells from Miltany Biotec (Bergisch Gladbach, Germany). cDNA was subsequently synthesized from 5 µg RNA by random priming, using Superscript II (Invitrogen).

Real-time quantitative expression analysis (RQ-PCR) was performed by the 7500 Fast Real-time System, using commercial buffer and primer sets (Applied Biosystems, Darmstadt, Germany). For normalization of expression levels we used TATA box binding protein (TBP). Quantitative analyses were performed in triplicate and repeated twice. The standard deviations are indicated in the figures as bars.

Reverse transcription (RT) PCR was performed using taqpol (Qiagen), oligonucleotides as listed in **Table S1** (MWG Eurofins, Martinsried, Germany) and the thermocycler TGradient (Biometra, Göttingen, Germany).

### Protein analysis

Sodium dodecyl sulphate polyacrylamid gel electrophoresis (SDS-PAGE) was performed in the Mini-Protean 3 system (Bio-Rad, München, Germany). Gels were either stained with PageBlue (Fermentas, Vilnius, Lithuania) or processed for Western blot analysis via the semi-dry method. Proteins obtained from cell lysates were transferred onto nitrocellulose membranes (Bio-Rad) and blocked with 5% dry milk powder dissolved in phosphate-buffered-saline buffer (PBS). The following antibodies were used: anti-NKX2-1 (EP1584Y, Abgent, Heidelberg, Germany), anti-NKX3.1 (clone 3–9, Invitrogen), anti-ERK1 (K-23, Santa Cruz Biotechnology, Heidelberg, Germany). The secondary antibodies were labelled with peroxidase and detected by Western Lightning-ECL (Perkin Elmer, Waltham, MA, USA).

### Chromatin immuno-precipitation (ChIP)

ChIP analysis was performed with the ChIP Assay Kit (Millipore-Upstate, Schwalbach, Germany) as described by the manufacturer, isolating genomic DNA fragments generated by sonication, using antibodies anti-NKX2-1 (EP1584Y, Abgent), anti-H2B (53H3, Cell Signaling, Danvers, MA, USA), anti-H2Bub1 (D11, Cell Signaling), anti-H3K4me3 (mAbcam1012, Abcam, Cambridge, UK) and anti-H3K27me3 (mAbcam6002, Abcam). The subsequent ChIP-PCR analysis was performed using taqpol (Qiagen) oligonucleotides as listed in **Table S1** (MWG Eurofins) and thermocycler TGradient (Biometra).

### Immunostaining

To determine intracellular localizations of proteins we performed immunostaining as described previously [23]. We used antibodies anti-NKX2-1 (EP1584Y, Abgent) and anti-SMAD3 (7F3, Abnova, Heidelberg, Germany). Secondary antibodies were purchased from Cell Signaling. For analysis cells were mounted with Vectashield (Vector Laboratories, Burlingame, CA, USA) containing the counterstain DAPI.

### Analysis of DNA methylation

SU-DHL-5 cells were treated with 10 µg/ml trichostatin A (TSA, Sigma) or 10 mM 5-Aza-2′-deoxycytidine (Sigma) for 20 h before being examined by RQ-PCR. To identify particular methylated cytidines within the CpG island at the HOPX locus, genomic DNA of SU-DHL-5 and SU-DHL-4 (for control) was subjected to bisulfite-conversion and analyzed as described recently [24]. The converted DNA was amplified by PCR using oligonucleotides as listed in **Table S1**. The PCR products were subcloned into pGEMT-easy (Promega, Madison, WI, USA) and the inserts sequenced (MWG Eurofins). Sequences of 10 clones were displayed and compared using BiQ Analyzer (http://biq-analyzer.bioinf.mpg.de).

## Results

### Ectopic expression of NKL homeobox gene NKX2-1 in DLBCL cell line

Here, we screened by expression profiling 20 leukemia/lymphoma T- and B-cell lines for aberrant activities of NKL-family homeobox genes which are implicated in T-cell leukemia [11]. Our findings confirmed expression of NKL family members in T-ALL cell lines, namely: TLX1 in ALL-SIL, TLX3 in HPB-ALL, NKX2-5 in PEER and CCRF-CEM, and NKX3-1 in JURKAT, PER-117 and RPMI-8402 (data not shown). In addition, we identified conspicuous expression of NKX2-1 and NKX3-1 in DLBCL cell line SU-DHL-5. Understanding the unexpected activation of NKL genes in a malignant B-cell line was the main focus of this enquiry.

**Figure 1:**
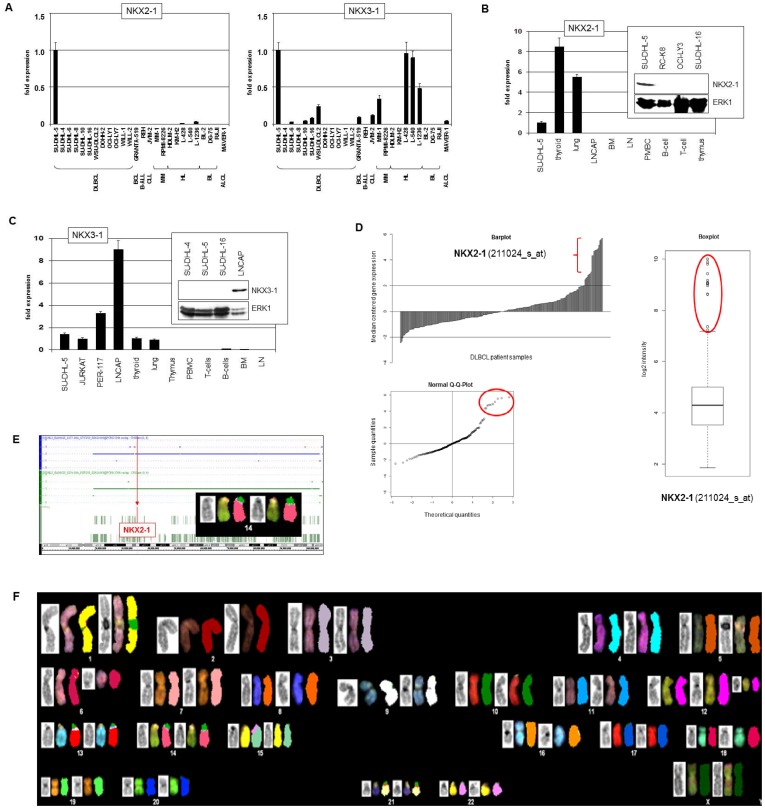
Expression and karyotyping. (A) RQ-PCR analysis of NKX2-1 (left) and NKX3-1 (right) in SU-DHL-5 (expression level was set to unity) and in leukemia/lymphoma control cell lines. (B) RQ-PCR analysis of NKX2-1 in primary cells, demonstrating physiological expression in thyroid and lung and ectopical expression in SU-DHL-5 cells. Western blot analysis (insert) confirms NKX2-1 expression in SU-DHL-5 cells while control DLBCL cell lines RC-K8, OCI-LY3 and SU-DHL-16 are negative. ERK1 was used as loading control. (C) RQ-PCR analysis of NKX3-1 in primary cells and selected cell lines, demonstrating physiological expression in thyroid, lung and prostate and ectopic expression in SU-DHL-5 and T-ALL cell lines JURKAT and PER-117. Western blot analysis (insert) shows NKX3-1 expression in prostate LNCAP cells while SU-DHL-5 lacks detectable NKX3-1 protein. (D) In silico expression analysis of NKX2-1 was performed in 204 DLBCL patient samples (NCBI GEO accession number GSE11318). Median centred gene expressions were illustrated in a barplot (left), showing 11 patients with enhanced expression of NKX2-1 (red bracket). Illustration of these datasets by a boxplot (right) and a Q-Q-plot (below) confirms significantly enhanced expression of NKX2-1 in these 11 patient samples (red circles). (E) Copy number analysis by genomic profiling indicates absence of aberrations at the locus of NKX2-1 at 14q13. The insert shows an enlargement of chromosome 14 obtained by SKY karyotyping. (F) SKY karyotyping of SU-DHL-5 shows absence of rearrangements at chromosome 14 while chromosome 6 demonstrates presence of aberrations.

Quantitative expression analysis of NKX2-1 and NKX3-1 by RQ-PCR confirmed their activation in SU-DHL-5 at the RNA level. While NKX3-1 was also expressed in Hodgkin lymphoma (HL), multiple myeloma (MM), B-cell lymphoma (BCL) and additional DLBCL cell lines, NKX2-1 was nearly undetectable in the same set of 25 lymphoma cell lines (**Fig. 1A**), prompting analysis of NKX2-1 and NKX3-1 in primary cells, including thyroid, lung and particular hematopoietic samples to chart physiological tissue specificity in the blood system. The results confirmed physiological NKX2-1 expression in tissues of the thyroid and the lung, while in all hematopoietic samples analyzed NKX2-1 transcription was undetectable (**Fig. 1B**). On the other hand, NKX3-1 expression was confirmed in the cell lines SU-DHL-5, JURKAT, PER-117 and LNCAP (prostate) and physiologically in tissues of the thyroid and the lung, while the hematopoietic samples of B-cells and BM showed just weak activity (**Fig. 1C**).

Protein expression of NKX2-1 and NKX3-1 was analyzed by Western blot. While NKX2-1 protein was clearly displayed in SU-DHL-5, NKX3-1 protein was not detectable in that cell line (**Fig. 1B,C**), consistent with post-transcriptional inhibition which was described recently [25]. For this reason we focused our work on regulation and function of homeobox gene NKX2-1, showing ectopic expression at the RNA and protein level in DLBCL cell line SU-DHL-5. To examine the expression of NKX2-1 in primary material we have checked 204 datasets of untreated DLBCL cases deposited in the GEO database of the NCBI belonging to the study of Lenz and coworkers [20]. This screening revealed statistically significant enhanced NKX2-1 activity in 11 (5%) DLBCL patients (**Fig. 1D**), supporting the relevance of this oncogenic homeobox gene expression in this malignancy. Of note, the NKX2-1 overexpressing DLBCL patients showed no clear correlation with known disease subsets.

Since deregulated expression of NKL homeobox genes in T-ALL is primarily caused by chromosomal aberrations, we analyzed the karyotype of SU-DHL-5 by SKY, FISH and in a genomic array with respect to the NKX2-1 gene which is located at 14q13 (36.9 Mb). However, copy number data (**Fig. 1E**), gene-specific FISH results (not shown) and SKY results (**Fig. 1F**) all returned wild type configurations at this locus, discounting a chromosomal mechanism behind the deregulated transcription.

Turning to potential transcriptional regulators which might induce aberrant NKX2-1 activity, we compared expression array data of SU-DHL-5 with 3 control DLBCL cell lines - SU-DHL-4, SU-DHL-10, and SU-DHL-16. After inspection of the top 1000 up- and downregulated genes in SU-DHL-5, potential candidates were shortlisted and functionally categorized as shown in **Table 1**. This exercise revealed conspicuous involvement of TFs, chromatin and signaling genes which were then subjected to more detailed consideration.

**Table 1:** Gene data of SU-DHL-5.

	Overexpressed	Downregulated	Mutated
**Transcription**	HEY1, NKX2-1, RUNX1T1, HOXC6, HOXA10, ETV5, NKX3-1, RUNX2, EGR1, POU2F2, TLE4, ZHX2	ETS2, TCL6, HLXB9, FLI1, LMO7, BCL11A	
**Chromatin**	HOPX, IGSF4, MLL, AF9, HIST1H3E	HMGN3, ASB2, JMJD1C, ARID5B, JMJD2B, PPP1R2, JARID1D, USP46, ARID4A	ARID1A, JARID2
**Signaling**	SMAD9, IL4, STAT3	SMAD6, IGF1R, BMPR2	IGF1R, MAP3K14, MAP2K1, MAP3K1, MAP3K4, MAP3K10, MAPK4
**NO/cAMP/cGMP/PDE**	DDAH1, PRKAR2B, PRKCE, NOS1	PDE3B, PDE4A, PDE6D, PDE9A, PRKAR2A, KCNQ1, AKAP7	AKAP12, PDE4DIP
**WNT-Signaling**	APCDD1, WNT5B, WNT5A, LEF1	CTNNA1, WNT3, VAV2, SOX4	
**NFkB-Signaling**	SOCS1, TNFSF11, TNFRSF21, PRKCE		
**Other**	DHRS2, EML1, ENO2	BCL2, CD99, STAG2	PTEN

Expression profiling data were analyzed and candidates among the top 1000 overexpressed and downregulated genes were selected and sorted according to their functional properties. Furthermore, mutated genes identified by sequence analysis of SU-DHL-5 (provided by the BROAD Institute, www.broadinstitute.org) were selected and sorted as well. The genes are categorized and ordered according their expression level or alphabetically for mutated genes.

### Transcription factors HEY1 and NKX2-1 are mutually exciting

**Figure 2:**
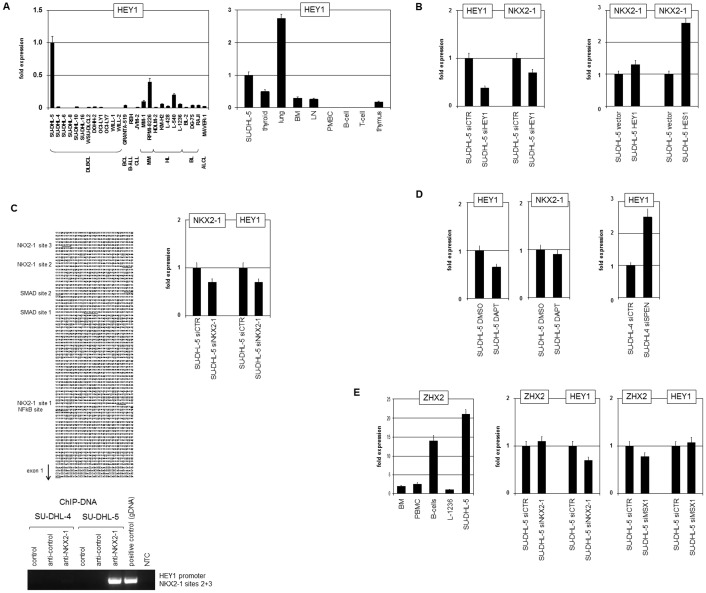
HEY1 and ZHX2. (A) RQ-PCR analysis of HEY1 in SU-DHL-5 (expression level was set to 1) and in leukemia/lymphoma control cell lines (left) and primary cells (right). (B) RQ-PCR analysis of SU-DHL-5 cells treated for siRNA-mediated knockdown of HEY1 (left) and for overexpression of HEY1 and HES1 (right). The results indicate activation of NKX2-1 by HEY1 (and HES1). (C) The promoter sequence of HEY1 contains binding sites for NKX2-1, SMAD and NFkB (left) which are highlighted in bold letters. SiRNA-mediated knockdown of NKX2-1 in SU-DHL-5 cells resulted in reduced expression of HEY1 as shown by RQ-PCR (right, above). ChIP analysis of the HEY1 promoter in SU-DHL-5 and SU-DHL-4 (for control) demonstrated direct binding of NKX2-1 as shown by PCR amplification of genomic fragments (left, below). Untreated genomic DNA served as positive control, NTC: no template control. (D) RQ-PCR analysis of HEY1 and NKX2-1 in SU-DHL-5 cells treated with NOTCH-inhibitor DAPT (left) or with siRNA directed against SPEN (right). (E) RQ-PCR analysis of ZHX2 in cell lines and primary cells (left), and of ZHX2 and HEY1 in siRNA-treated SU-DHL-5 cells (middle and right).

Among TFs bHLH factor HEY1 was particularly intriguing (one of the top 10 upregulated genes). RQ-PCR analysis of a panel of cell lines confirmed high expression levels in SU-DHL-5 (**Fig. 2A**). However, MM and HL cell lines also showed moderate transcription of HEY1. RQ-PCR analysis of SU-DHL-5 in comparison to primary cells revealed prominent HEY1 expression in lung, thyroid, and in BM, LN and thymus. These data suggest a functional role of HEY1 in early lymphopoiesis. Peripheral B-cells and PBMCs lacked HEY1 transcripts (**Fig. 2A**), indicating downregulation in differentiated B-cells and other leukocytes and thus aberrant upregulation in SU-DHL-5.

To analyze the regulatory impact of HEY1 on NKX2-1 expression we treated SU-DHL-5 cells with siRNA directed against HEY1. Subsequent quantification of HEY1 and NKX2-1 expression demonstrated reduction of both transcripts as compared to cell samples treated with control siRNA (**Fig. 2B**). Furthermore, overexpression of HEY1 or the related TF HES1 was followed by increased NKX2-1 expression (**Fig. 2B**). Together, these results show that HEY1 contributes to NKX2-1 expression in DLBCL cell line SU-DHL-5. That HEY1 acts as a transcriptional repressor suggests indirect activation of NKX2-1 probably via inhibition of negative regulators as shown below [26].

Sequence analysis of the promoter region of HEY1 identified 3 potential binding sites for NKX2-1 (**Fig. 2C**), indicating direct regulatory impact of this homeoprotein in HEY1 expression. Subsequent siRNA-mediated knockdown of NKX2-1 inhibited transcription of NKX2-1 and HEY1, confirming regulation by NKX2-1 (**Fig. 2C**). ChIP analysis using anti-NKX2-1 confirmed direct binding of NKX2-1 to the promoter region of HEY1 (**Fig. 2C**). These data show that NKX2-1 activates HEY1 transcription directly. Genomic copy number data and SKY analyses excluded genomic aberrations at the HEY1 locus at 8q21 (**Fig. S1**), highlighting the role of NKX2-1 in HEY1 regulation.

HEY1 and HES1 are prominent targets of the NOTCH-pathway in lymphopoiesis [4]. To analyze the potential impact of NOTCH on HEY1 expression we treated SU-DHL-5 cells with the γ-secretase inhibitor DAPT. Subsequent RQ-PCR analysis showed reduced HEY1 levels in treated samples (**Fig. 2D**), confirming NOTCH regulation. Accordingly, siRNA-mediated knockdown of NOTCH corepressor SPEN enhanced HEY1 expression more than twofold (**Fig. 2D**). Additional findings using DAPT discounted regulation of NKX2-1 by NOTCH-signaling (**Fig. 2D**).

Expression of Zn-finger homeobox gene 2 (ZHX2) showed elevated levels in SU-DHL-5 as well (**Table 1**). Comparative RQ-PCR analysis confirmed high transcript levels in SU-DHL-5, even surpassing primary B-cells (**Fig. 2E**). Of note, we recently showed reduced expression of ZHX2 in HL cell line L-1236 and an activating input of homeobox gene MSX1 [27]. Accordingly, siRNA-mediated knockdown of MSX1 in SU-DHL-5 reduced transcription of ZHX2 but not of HEY1 (**Fig. 2E**), contrasting with stimulation of HEY1 by the closely related homeobox gene MSX2 in T-ALL cells [28]. SiRNA-mediated knockdown of homeobox gene NKX2-1 reduced transcript levels of HEY1 as shown above while sparing ZHX2 (**Fig. 2E**), thus discounting direct regulation of ZHX2 by NKX2-1. However, the precise mechanism of ZHX2 enhancement remains unclear.

### Aberrant chromatin structures mediate NKX2-1 expression

**Figure 3:**
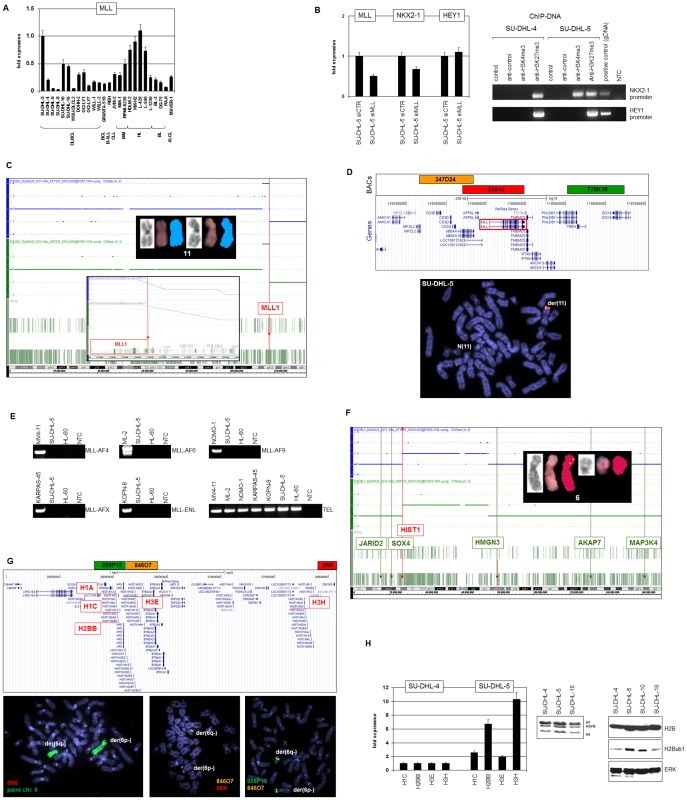
MLL and HIST1. (A) RQ-PCR analysis of MLL in SU-DHL-5 (expression level was set to unity) and in leukemia/lymphoma control cell lines. (B) RQ-PCR analysis of MLL, NKX2-1 and HEY1 in siRNA-treated SU-DHL-5 cells (left). ChIP analysis of the NKX2-1 and HEY1 promoters in SU-DHL-5 and SU-DHL-4 (for control) showed representation of particular histone H3 modifications, as shown by PCR amplification of genomic fragments (right). Untreated genomic DNA served as positive control, NTC: no template control. (C) Copy number analysis by genomic profiling indicates presence of aberrations at MLL at 11q23. Inserts show an enlargement of chromosome 11 obtained by SKY karyotyping and an enlargement of the MLL locus obtained by genomic profiling. (D) FISH analysis of the MLL locus in SU-DHL-5 (below) using BAC probes as indicated above. The results show one wild type allele and one amplified MLL locus. (E) RT-PCR analysis of MLL fusion genes in SU-DHL-5 and particular positive and negative control cell lines. TEL expression served as control, NTC: no template control. (F) Copy number analysis by genomic profiling of chromosome 6 indicates extended deletions at both arms. The HIST1 locus maps to the breakpoint region at 6p22. Genes located in deleted regions include JARID2, SOX4, HMGN3, AKAP7 and MAP3K4. Insert shows chromosomes 6 analyzed by SKY karyotyping indicating rearrangements at both chromosomes. (G) FISH analysis of the histone gene cluster HIST1 at 6p22 (below) using painting probe and BACs as indicated above. (H) RQ-PCR analysis of selected histone genes in SU-DHL-5 and SU-DHL-4 for control (left). PAGE analysis of histone proteins in three DLBCL cell lines (middle) demonstrates elevated levels in SU-DHL-5. Western blot analysis of H2B, H2Bub1 and ERK (for control) in four DLBCL cell lines (right) demonstrates elevated levels in SU-DHL-5.

The catalogue of upregulated genes in SU-DHL-5 encoding chromatin components included prominently MLL which is frequently deregulated in leukemia where it activates homeobox genes of the clustered type via H3K4-trimethylation [16]. Quantification of MLL expression in cell lines confirmed elevated RNA levels in SU-DHL-5 in addition to HL cells (**Fig. 3A**). SiRNA-mediated knockdown of MLL inhibited expression of NKX2-1 but not of HEY1 (**Fig. 3B**), showing that MLL supports NKX2-1 expression. To analyze corresponding histone modifications at the promoter-regions of NKX2-1 and HEY1 we performed ChIP using antibodies for activatory H3K4me3 (mediated by MLL) and inhibitory H3K27me3 (mediated by EZH2). Analysis of the NKX2-1 promoter showed presence of H3K27me3 in both SU-DHL-4 and SU-DHL-5, while H3K4me3 was restricted to SU-DHL-5 (**Fig. 3B**). The HEY1 promoter exclusively bore H3K27me3 in both cell lines (**Fig. 3B**). The presence of both types of analyzed H3-trimethylations at the NKX2-1 locus in SU-DHL-5 indicates aberrant bivalent chromatin configuration known to prime developmental genes for activation and thus likely to favor NKX2-1 expression in this cell line [29]. Moreover, profiling data indicated overexpression of the MLL complex component AF9 and reduced expression of ASB2 which mediates degradation of MLL (**Table 1**) [17], [30].

In leukemia MLL is frequently activated by chromosomal aberrations at 11q23 resulting in amplifications or diverse fusion genes [31]. We looked for chromosomal rearrangements in SU-DHL-5 by genomic profiling, finding duplication of MLL accompanied by deletion of its immediately telomeric region (**Fig. 3C**). FISH analysis confirmed that MLL gain was coupled with the downstream deletion (**Fig. 3D**). SKY results excluded chromosomal translocations at 11q23 (**Fig. 1D**), and RT-PCR analysis of the most prolific MLL-fusion transcripts excluded cryptic fusions with AF4, AF6, AF9, AFX and ENL (**Fig. 3E**). Collectively, our results show that genomic copy number gain of wild type MLL underlies overexpression of MLL in SU-DHL-5 cells.

Furthermore, overexpression of histone H3E (**Table 1**) correlated with rearrangements of chromosome 6 targeting histone gene cluster 1 (HIST1) at 6p22 as indicated by genomic array data (**Fig. 3F**). FISH analysis using probes covering HIST1 combined with a painting probe for chromosome 6 confirmed the breakpoint nearby, lying nevertheless outwith the gene cluster (**Fig. 3G**). The FISH results are consistent with the SKY data showing two derivative chromosomes 6 harboring deletions at 6p22 and 6q13, respectively (**Fig. 1D** **,** **3F**). Quantification of several histone RNA species demonstrated abundant expression of H1C, H2BB, H3E and H3H in SU-DHL-5 as compared to SU-DHL-4 (**Fig. 3H**), consistent with coordinate gene activation at the histone locus at 6p22 by nearby chromosomal rearrangement. Accordingly, SU-DHL-5 contained higher levels of core-histone proteins as shown by SDS-PAGE (**Fig. 3H**). Moreover, histone H2B when analyzed by Western blot showed raised protein expression and enhanced modification with a single ubiquitin at lysine 120, subsequently named H2Bub1 (**Fig. 3H**). The latter is of special interest because H2Bub1 reportedly promotes trimethylation of H3K4 by MLL [32], suggesting collaborative activities of the chromosomal aberrations targeting MLL and HIST1.

**Figure 4:**
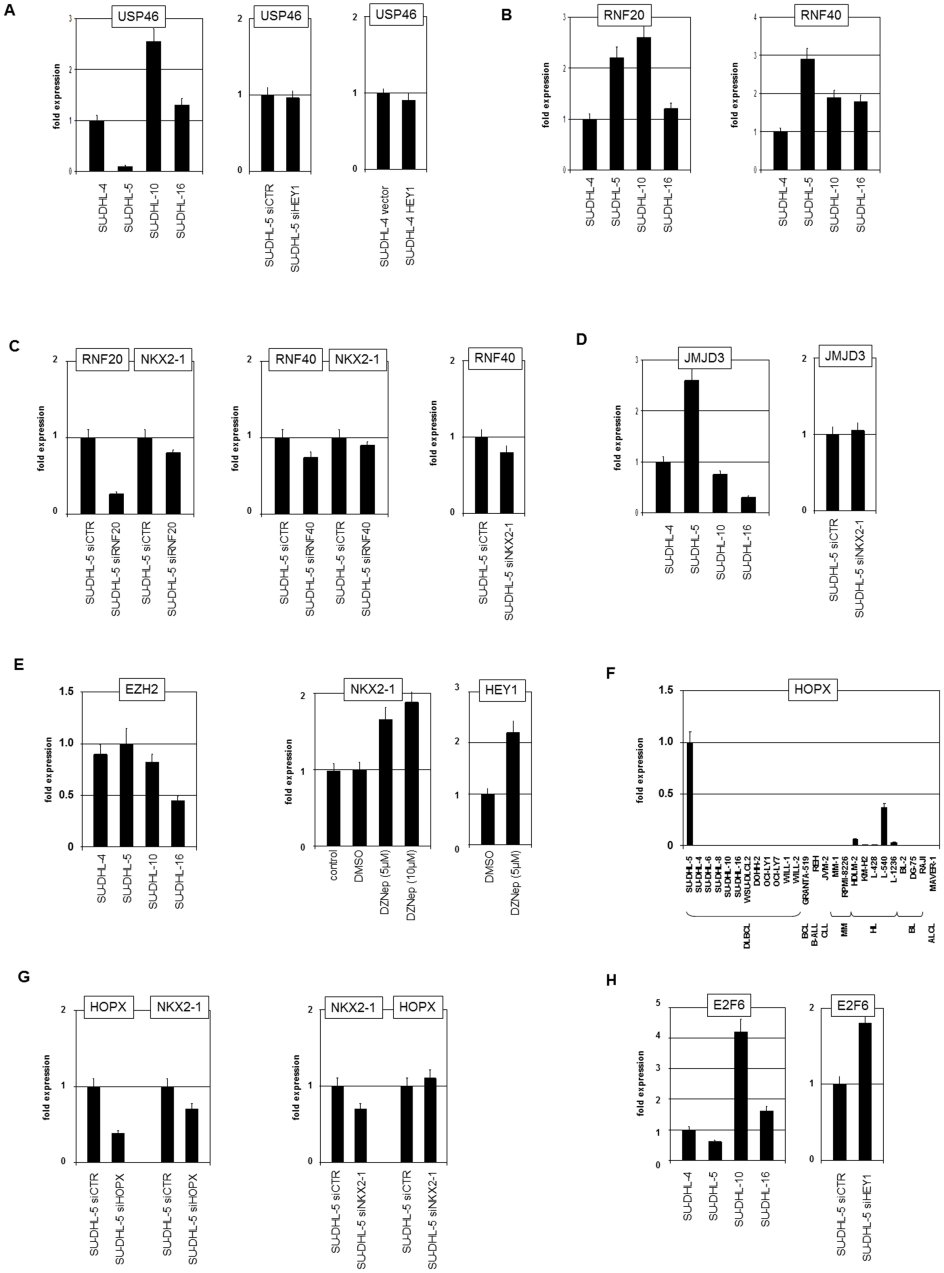
Signaling pathways. (A) RQ-PCR analysis of NKX2-1 and HEY1 in SU-DHL-5 cells treated with TNFa for 1 h (left) and 4 h (right). (B) RQPCR analysis of NKX2-1 and HEY1 in SU-DHL-5 cells treated with NFkB-inhibitor for 4 h (left) and 16 h (right). (C) RQ-PCR analysis of PRKCE in DLBCL cell lines (left) and in SU-DHL-5 cells treated for siRNA-mediated knockdown of NKX2-1 (middle). ChIP analysis of the PRKCE promoter in SU-DHL-5 and SU-DHL-4 (for control) demonstrated binding of NKX2-1 as shown by PCR amplification of genomic fragments (right, below). Untreated genomic DNA served as positive control, NTC: no template control. (D) RQ-PCR analysis of NKX2-1 and HEY1 in SU-DHL-5 cells treated with TGFb for 1 h (left) and 16 h (right). (E) RQ-PCR analysis of NKX2-1 and HEY1 in SU-DHL-5 cells treated with inhibitory antibody directed against TFGb for 16 h showed no significant differences. (F) RQ-PCR analysis of NKX2-1 and HEY1 in SU-DHL-5 cells treated with siRNA directed against SMAD3 and SMAD9 (above). Immuno-fluorescence analysis of SMAD3 (green) and NKX2-1 (red) in SU-DHL-5 demonstrates nuclear colocalization as compared to the DAPI-stained (blue) nucleus (below). (G) RQ-PCR analysis of NKX2-1 (left) and HEY1 (middle) in SU-DHL-5 cells treated with IL4, BMP4, IL10 and WNT5B for 16 h. RQPCR analysis of NKX2-1 and HEY1 in SU-DHL-5 cells treated for siRNA-mediated knockdown of STAT3 (right). (H) Heatmap of PDE genes in 6 DLBCL cell lines. PDE3B, PDE4A, PDE4B, PDE6D and PDE9A are highlighted. Red indicates high expression levels, green low, and black medium levels. (I) RQPCR analysis of NKX2-1 and HEY1 in SU-DHL-5 cells treated with cAMP, cGMP and sildenafil for 4 h and 16 h. (K) RQ-PCR analysis of NKX2-1 and HEY1 in SU-DHL-5 cells treated for siRNA-mediated knockdown of PDE6D (left). RQ-PCR analysis of NOS1 in DLBCL cell lines (middle) and in SU-DHL-5 cells treated for siRNA-mediated knockdown of NKX2-1 (right). (L) RQ-PCR analysis of PDE4A and PDE6D in SU-DHL-5 cells treated for siRNA-mediated knockdown of HEY1.

H2B ubiquitinylation levels are regulated by counteracting ubiquitin-transferases (RNF20, RNF40) and ubiquitin-specific proteases (USPs) [33]. Profiling data indicated repression of USP46 in SU-DHL-5 as confirmed by RQ-PCR analysis in comparison to control cell lines (**Fig. 4A**). However, USP46 expression was not regulated by the transcriptional repressor HEY1, as analyzed by knockdown and overexpression experiments (**Fig. 4A**). RQ-PCR analysis of RNF20 and RNF40 showed upregulation in SU-DHL-5 relative to control cell lines (**Fig. 4B**). Knockdown of RNF20 and RNF40 by siRNA treatment inhibited expression of NKX2-1 (**Fig. 4C**) demonstrating that these H2B ubiquitin-transferases support expression of that homeobox gene. Moreover, siRNA-mediated knockdown of NKX2-1 inhibited expression of RNF40 (**Fig. 4C**), showing that expression of RNF40 is supported by NKX2-1 and thus presence of reciprocal regulation.

These results prompted us to look for additional deregulated histone modifiers which may contribute to the aberrant chromatin structure at NKX2-1. Inhibitory trimethylation of H3K27 is conducted by polycomb repressor complex (PRC) 2 (containing EZH2, JARID2, HOPX, E2F6), and is removed by histone methylase JMJD3 [34], [35]. The expression level of JMJD3 was elevated in SU-DHL-5 as shown by RQ-PCR analysis (**Fig. 4D**). However, JMJD3 was not regulated by NKX2-1 (**Fig. 4D**). The expression of EZH2 was not significantly altered in SU-DHL-5 as compared to control cell lines (**Fig. 4E**). Nevertheless, treatment of SU-DHL-5 with EZH2/PRC2 inhibitor DZNep resulted in enhanced transcription of both NKX2-1 and HEY1 (**Fig. 4E**). These results are consistent with our ChIP data, showing presence of EZH2-mediated H3K27me3 at the promoter regions of both genes (**Fig. 3B**). Although microarray expression of the gene encoding PRC2 component JARID2 was normal, genomic array data showed monoallelic deletion at 6p22 (**Fig. 3F**). Sequence data of SU-DHL-5 cells (provided by the BROAD Institute, **Table 1**) showed mutation of JARID2. Thus SU-DHL-5 is hemizygous for mutated JARID2. Homeobox only protein (HOPX) is associated with PRC2, regulating its capacity for repression [36], [19]. SU-DHL-5 showed remarkably high levels of HOPX expression as indicated by profiling data (**Table 1**) and confirmed by RQ-PCR results of lymphoma cell lines (**Fig. 4F**). SiRNA-mediated knockdown of HOPX resulted in reduced expression of NKX2-1 (**Fig. 4G** **)**, showing that HOPX activates NKX2-1 transcription. But this activation was not reciprocal, since reduction of NKX2-1 was unaccompanied by altered HOPX levels (**Fig. 4G**). However, genomic copy number data and SKY results excluded genomic aberrations at the HOPX locus (**Fig. S2**). Moreover, examination of DNA methylation of a conspicuous CpG island at the HOPX locus (CpG 109) excluded an abnormal configuration (**Fig. S3**), leaving the mechanism of this striking overexpression elusive. HOPX is associated with E2F6 which showed reduced expression levels in SU-DHL-5 (**Fig. 4H**). Interestingly, this reduction was mediated by HEY1 as shown by knockdown experiments which led to overexpression of E2F6 (**Fig. 4H**). Together, our results demonstrate a significant role for several chromatin modifiers underlying NKX2-1 activation in SU-DHL-5. These regulatory interactions are partly reciprocal and constitute feedback-loops, probably resulting in enhanced and stabilized gene activities.

### Signaling pathways differentially regulate expression of NKX2-1 and HEY1

The results of comparative expression analysis in SU-DHL-5 indicated deregulation of several signaling components belonging to a variety of pathways, including TNFa/NFkB/PRKC, TGFb/BMP/SMAD, IL4/STAT3 and NOS1/cAMP/cGMP/phosphodiesterases (PDEs) which plausibly contribute to NKX2-1 and/or HEY1 expression (**Table 1**). These pathways were analyzed in subsequent experiments.

**Figure 5:**
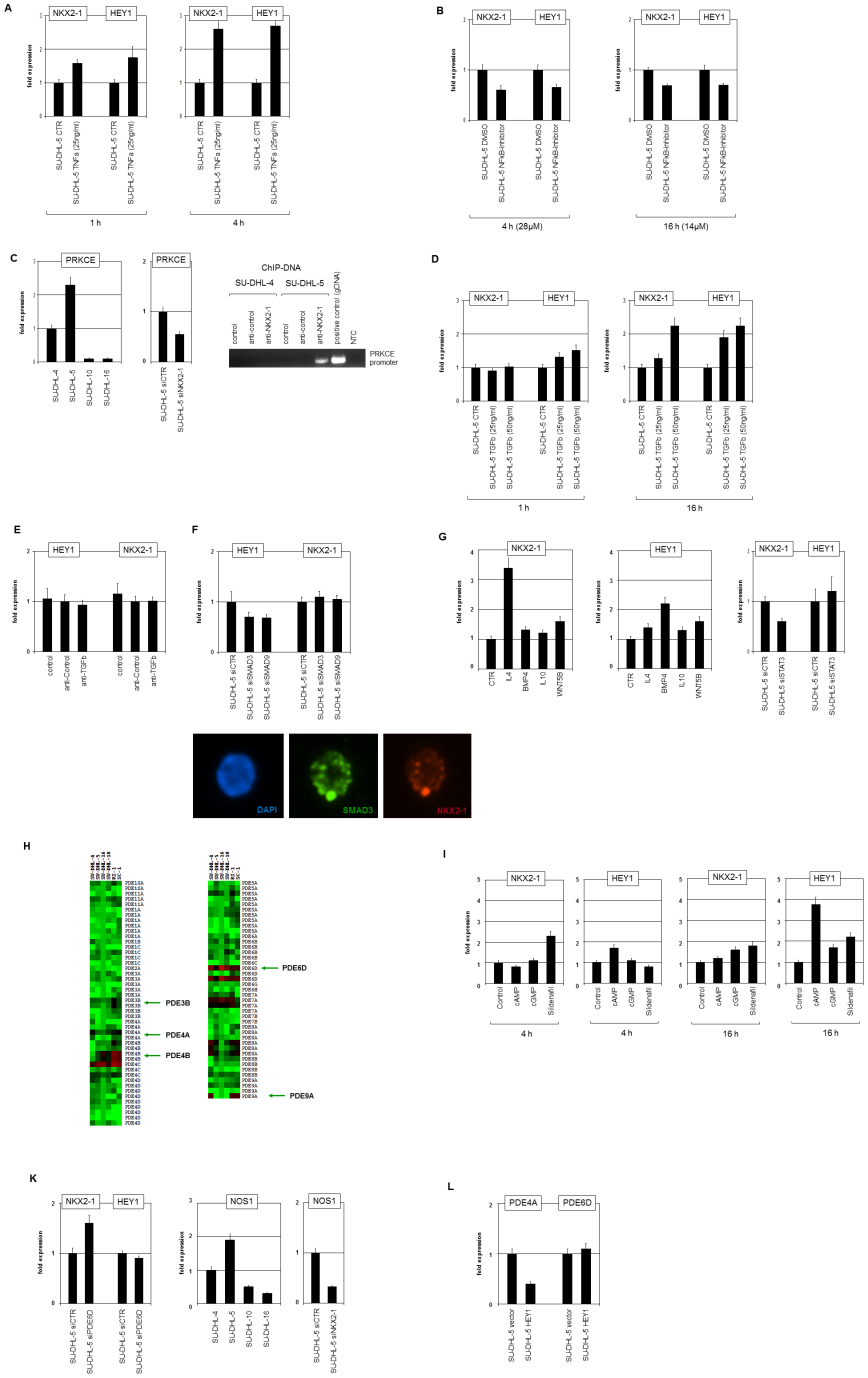
Signaling pathways. (A) RQ-PCR analysis of NKX2-1 and HEY1 in SU-DHL-5 cells treated with TNFa for 1 h (left) and 4 h (right). (B) RQ-PCR analysis of NKX2-1 and HEY1 in SU-DHL-5 cells treated with NFkB-inhibitor for 4 h (left) and 16 h (right). (C) RQ-PCR analysis of PRKCE in DLBCL cell lines (left) and in SU-DHL-5 cells treated for siRNA-mediated knockdown of NKX2-1 (middle). ChIP analysis of the PRKCE promoter in SU-DHL-5 and SU-DHL-4 (for control) demonstrated binding of NKX2-1 as shown by PCR amplification of genomic fragments (right, below). Untreated genomic DNA served as positive control, NTC: no template control. (D) RQ-PCR analysis of NKX2-1 and HEY1 in SU-DHL-5 cells treated with TGFb for 1 h (left) and 16 h (right). (E) RQ-PCR analysis of NKX2-1 and HEY1 in SU-DHL-5 cells treated with inhibitory antibody directed against TFGb for 16 h showed no significant differences. (F) RQ-PCR analysis of NKX2-1 and HEY1 in SU-DHL-5 cells treated with siRNA directed against SMAD3 and SMAD9 (above). Immuno-fluorescence analysis of SMAD3 (green) and NKX2-1 (red) in SU-DHL-5 demonstrates nuclear colocalization as compared to the DAPI-stained (blue) nucleus (below). (G) RQ-PCR analysis of NKX2-1 (left) and HEY1 (middle) in SU-DHL-5 cells treated with IL4, BMP4, IL10 and WNT5B for 16 h. RQ-PCR analysis of NKX2-1 and HEY1 in SU-DHL-5 cells treated for siRNA-mediated knockdown of STAT3 (right). (H) Heatmap of PDE genes in 6 DLBCL cell lines. PDE3B, PDE4A, PDE4B, PDE6D and PDE9A are highlighted. Red indicates high expression levels, green low, and black medium levels. (I) RQ-PCR analysis of NKX2-1 and HEY1 in SU-DHL-5 cells treated with cAMP, cGMP and sildenafil for 4 h and 16 h. (K) RQ-PCR analysis of NKX2-1 and HEY1 in SU-DHL-5 cells treated for siRNA-mediated knockdown of PDE6D (left). RQ-PCR analysis of NOS1 in DLBCL cell lines (middle) and in SU-DHL-5 cells treated for siRNA-mediated knockdown of NKX2-1 (right). (L) RQ-PCR analysis of PDE4A and PDE6D in SU-DHL-5 cells treated for siRNA-mediated knockdown of HEY1.

Treatment of SU-DHL-5 cells with TNFa for 1 h or 4 h resulted in activated transcription of both NKX2-1 and HEY1 (**Fig. 5A**). In accordance with this result, treatment with NFkB inhibitor reduced expression of both genes (**Fig. 5B**), demonstrating a positive role for TNFa/NFkB-signaling in transcriptional regulation. Furthermore, expression of protein kinase C variant E (PRKCE) was enhanced in SU-DHL-5 when compared to control cell lines (**Table 1**, **Fig. 5C**). This may be of interest because the activity of NFkB is regulated by PRKC. SiRNA-mediated reduction of NKX2-1 was accompanied by downregulation of PRKCE (**Fig. 5C**), indicating a positive regulatory role for this NKL homeobox gene. Sequence analysis of the upstream region of PRKCE (UCSC genome browser, release GRCh37/hg19) revealed a binding site for NKX2-1 at -25.832 bp. ChIP analysis of this site confirmed binding of NKX2-1 antibody, demonstrating direct activation of PRKCE by NKX2-1 (**Fig. 5C**). This kind of gene regulation represents positive feedback activation which activates involved genes, demonstrating a complex gene regulatory network contributing to ectopic NKX2-1 expression.

Treatment of SU-DHL-5 cells with TGFb resulted in enhanced transcription of both NKX2-1 and HEY1 after 16 h (**Fig. 5D**). Interestingly, after 1 h of treatment the expression of HEY1 raised concentration-dependend while NKX2-1 showed no change in transcript levels at this time point (**Fig. 5D**). These results may indicate that HEY1 is a direct target of TGFb-signaling, contrasting with the delayed and thus indirect mechanism of NKX2-1 regulation. However, treatment of SU-DHL-5 with inhibitory anti-TGFB showed no effect, excluding autocrine activation (**Fig. 5E**). SiRNA-mediated knockdown of SMAD3 or SMAD9 reduced transcription of HEY1 while that of NKX2-1 remained unperturbed (**Fig. 5F**), supporting the direct regulation of HEY1 by TGFb/SMAD-signaling. Of note, according to the UCSC genome browser (release GRCh37/hg19) the promoter-region of HEY1 contains binding-sites for SMAD proteins which are colocated with those of NKX2-1 (**Fig. 2C**): a significant observation, since SMAD3 protein has been shown to interact with NKX2-1 [37]. Immunostaining of NKX2-1 and SMAD3 in SU-DHL-5 cells consistently revealed colocalization of both TFs (**Fig. 5F**). Therefore, our results indicate that in SU-DHL-5 cells NKX2-1 and SMAD3 coactivate HEY1 by protein-protein interaction and direct binding to the promoter region.

The impact of additional pathways in expression of NKX2-1 and HEY1 was analyzed by treatment of SU-DHL-5 cells with IL4, BMP4, IL10 and WNT5B for 16 h (**Fig. 5G**). The most significant effects on NKX2-1 were observed after stimulation with IL4 and for HEY1 with BMP4. BMP4 signaling is mediated by SMAD proteins just like TGFb, showing consistent results for HEY1 regulation. IL4-signaling is mediated by STAT3 which is upregulated in SU-DHL-5 (**Table 1**). Accordingly, siRNA-mediated knockdown of STAT3 reduced expression of NKX2-1 while HEY1 transcription was not significantly affected (**Fig. 5G**). These results support an activating role for IL4/STAT3-signaling on NKX2-1 and for BMP4/SMAD-signaling on HEY1 in SU-DHL-5.

Finally, we analyzed the impact of cAMP/cGMP-signaling in expression of NKX2-1 and HEY1. This pathway stood out due to overexpression of NOS1 (synthesizes nitric oxide activating guanylate cyclase) and reduced expression levels of several PDEs as indicated by comparative profiling data (**Table 1**) and illustrated by a heatmap for PDE-expression (**Fig. 5H**). Furthermore, overexpressed DDAH1 (**Table 1**) encodes an inhibitor of the negative regulator ADMA for NOS1. Therefore, SU-DHL-5 cells were treated with cAMP, cGMP and cGMP-specific PDE-inhibitor sildenafil and subsequently quantified for NKX2-1 and HEY1 transcription (**Fig. 5I**). After 4 h expression of NKX2-1 rose significantly after treatment with sildenafil, while HEY1 rose with cAMP. After 16 h expression of NKX2-1 peaked in response to treatment with sildenafil and cGMP, while HEY1 responded maximally to treatment with cAMP and to a lesser extent with cGMP and sildenafil (**Fig. 5I**). These data suggest that expression of NKX2-1 and HEY1 is primarily regulated via cGMP and cAMP, respectively. Accordingly, siRNA-mediated knockdown of cGMP-specific PDE6D resulted in enhanced expression of NKX2-1 while HEY1 remained unchanged (**Fig. 5K**). The enhanced expression of NOS1 in SU-DHL-5 as compared to control cell lines was confirmed by RQ-PCR analysis (**Fig. 5K**). SiRNA-mediated knockdown of NKX2-1 resulted in strong reduction of NOS1, indicating an activatory role for NKX2-1 (**Fig. 5K**). However, ChIP analysis excluded direct binding of NKX2-1 to the promoter region of NOS1 (data not shown), suggesting an indirect activation mechanism. Finally, the transcriptional repressor HEY1 was found to underly PDE4A repression, while non-participant in PDE6D regulation, as shown by overexpression experiments (**Fig. 5L**). Thus, deregulated expression of PDEs (PDE6D, PDE4A) and NOS1 via HEY1 and NKX2-1 indicate feedback regulation of both TFs.

## Discussion

In DLBCL cell line SU-DHL-5 we have identified ectopic expression of NKX2-1 which is activated by bHLH TF HEY1, aberrant modifications of the chromatin structure, and particular signaling pathways. NKX2-1 belongs to the NKL family of homeobox genes which is implicated in the tumorigenesis of T-ALL [11], [38]. In silico expression analysis of patient samples indicated aberrant activity of NKX2-1 in 5% of DLBCL, representing a hitherto unrecognized subgroup of this disease. Therefore, our results expand the oncogenic role of this gene family within the entity of lymphoid malignancies.

NKX2-1 is physiologically expressed in the developing lung and thyroid but not, as shown here, in hematopoietic cells. In a physiological context NKX2-1 regulates differentiation processes both during embryogenesis and in the adult [7], [8]. In lung cancer NKX2-1 performs the role of a lineage-specific oncogene enhancing proliferation and survival [39], [40]. Overexpression of NKX2-1 mediated by genomic amplification enhances tumorigenicity of lung cancer cells as evidenced by colony formation of lung epithelial cells and advanced malignancy in affected patients [41]. Furthermore, NKX2-1 enhances together with FOXA1 survival in lung adenocarcinoma by transcriptional activation of LMO3 [42]. However, we have neither experimentally assayed tumorigenicity, nor survival of SU-DHL-5 cells, but our comparative expression data gave no hint for deregulation of proliferation or apoptosis. Accordingly, SU-DHL-5 showed no increased expression of LMO3, suggesting absence of this particular survival-pathway. Rather, the profiling data of SU-DHL-5 identified TFs and signaling pathways highlighting the view of deregulated cell differentiation mediated by NKX2-1.

Our data ruled out aberrant activation of NKX2-1 via chromosomal rearrangements contrasting with the picture of NKL homeo-oncogenes in T-ALL. We identified several (deregulated) genes involved in NKX2-1 expression by comparative profiling and subsequent knockdown and overexpression studies. Among TFs we identified activating HEY1 which underlies NKX2-1 transcription. HEY1 belongs to the inhibitory subgroup of bHLH proteins, deregulation of which promotes the development of leukemia/lymphoma affecting the function of E2A in driving lymphoid development [26], [36], [43]-[45]. Our data show direct activation of HEY1 by NKX2-1 and indirect activation of NKX2-1 by repressive HEY1. HEY1 is physiologically expressed in developing lung tissue like NKX2-1 [7], [46]. Therefore, this regulatory role may also figure in the physiological context of the lung. However, forced expression of HEY1 in DLBCL cell lines did not induce NKX2-1 transcription, indicating that additional factors or chromatin modifications are necessary for the gene activity as described below.

MLL contributes to enhanced expression of NKX2-1 in SU-DHL-5 cells. It is overexpressed in this cell line via chromosomal rearrangements resulting in duplication of the wild type gene. Tandem triplication of MLL has been described in intravascular large B-cell lymphoma suggesting a more widespread oncogenic role in B-cell lymphomas than hitherto supposed [47]. The MLL gene encodes a methyltransferase which modifies histone H3 (H3K4me3). This modification marks active chromatin and gene transcription [17]. The presence of both activatory H3K4me3 and inhibitory H3K27me3 as detected here in SU-DHL-5 at NKX2-1 has been termed “bivalent chromatin modification”–a structure which primes developmental genes for activation in embryonal stem cells [29]. Therefore, this histone-mark may represent one of the basic factors predisposing to ectopic NKX2-1 activation.

Additionally, overexpression of core-histones in SU-DHL-5 coincided with a chromosomal aberration at 6p22 housing the histone gene cluster 1. H2B in particular was shown to be overexpressed and strongly ubiquitinated at position K120. This modification guides and enhances the process of MLL-mediated H3K4-methylation, indicating cooperation of both types of chromosomal rearrangements in NKX2-1 expression in SU-DHL-5 [32].

Several enzymes performing histone-modifications were deregulated in SU-DHL-5, contributing to a permissive chromatin structure at the NKX2-1 gene. RNF and USP genes encode ubiquitin-specific transferases and proteases, respectively, regulating ubiquitination of histone H2B [33]. Our results demonstrate, in addition to aberrant expression, their impact on deregulating NKX2-1 transcription. Accordingly, NKX2-1 has been described as a target gene of RNF20 in HELA cells [48]. PRC2 contains H3-methyltransferase EZH2 and the modulating components HOPX and E2F6 [49], [50]. Expression levels of HOPX and E2F6 were altered in SU-DHL-5 and functional analyses demonstrated their impact in NKX2-1 regulation. However, while HOPX expression is activated by NKX2-1 in lung cancer cells, it was not regulated by NKX2-1 in SU-DHL-5 cells [51]. Of note, both HOPX and E2F6 are overexpressed in HL indicating the presence therein of deregulated chromatin structures, albeit distinct from those in SU-DHL-5 [52], [23]. Interestingly, in SU-DHL-5 many genes encoding deregulated histone modifiers are influenced by NKX2-1 or HEY1 in their expression levels, revealing a reciprocal network which mutually reinforces aberrant oncogene activities.

As well as TFs and chromatin-modifiers we identified signaling pathways regulating NKX2-1 expression: firstly, TNFa, NFkB and the NFkB-activating kinase PRKCE were involved in activation of both NKX2-1 and HEY1; second, IL4/STAT3-signaling which was primarily engaged in the activation of NKX2-1; and finally, TGFb/BMP4 and SMAD3 which activated transcription of just HEY1. This last named activity may explain both the presence of neighboring binding sites for NKX2-1 and SMAD seen at the HEY1-promoter, and nuclear colocalization of NKX2-1 and SMAD3 in SU-DHL-5, indicating cooperative activation of HEY1. This coactivation may represent a switch-like regulation which stabilizes gene activities [53]. Furthermore, enzymes regulating levels of cGMP and cAMP were identified as respective mediators of NKX2-1 and HEY1 expression, including NOS1 and specific PDEs. Mutated genes of this pathway in SU-DHL-5 include PDE4DIP and AKAP12. Of note, while reduced levels of PDEs were identified here in a DLBCL cell line, enhanced levels of PDE5A have been reported in HL cells [54], suggesting that PDE-activity may be critical for lymphomagenesis. Furthermore, sequence data revealed several mutated MAP kinases, e.g. MAP3K14, MAP2K1, and MAPK4. However, their impact in NKX2-1 expression was not considered in this study.

**Figure 6:**
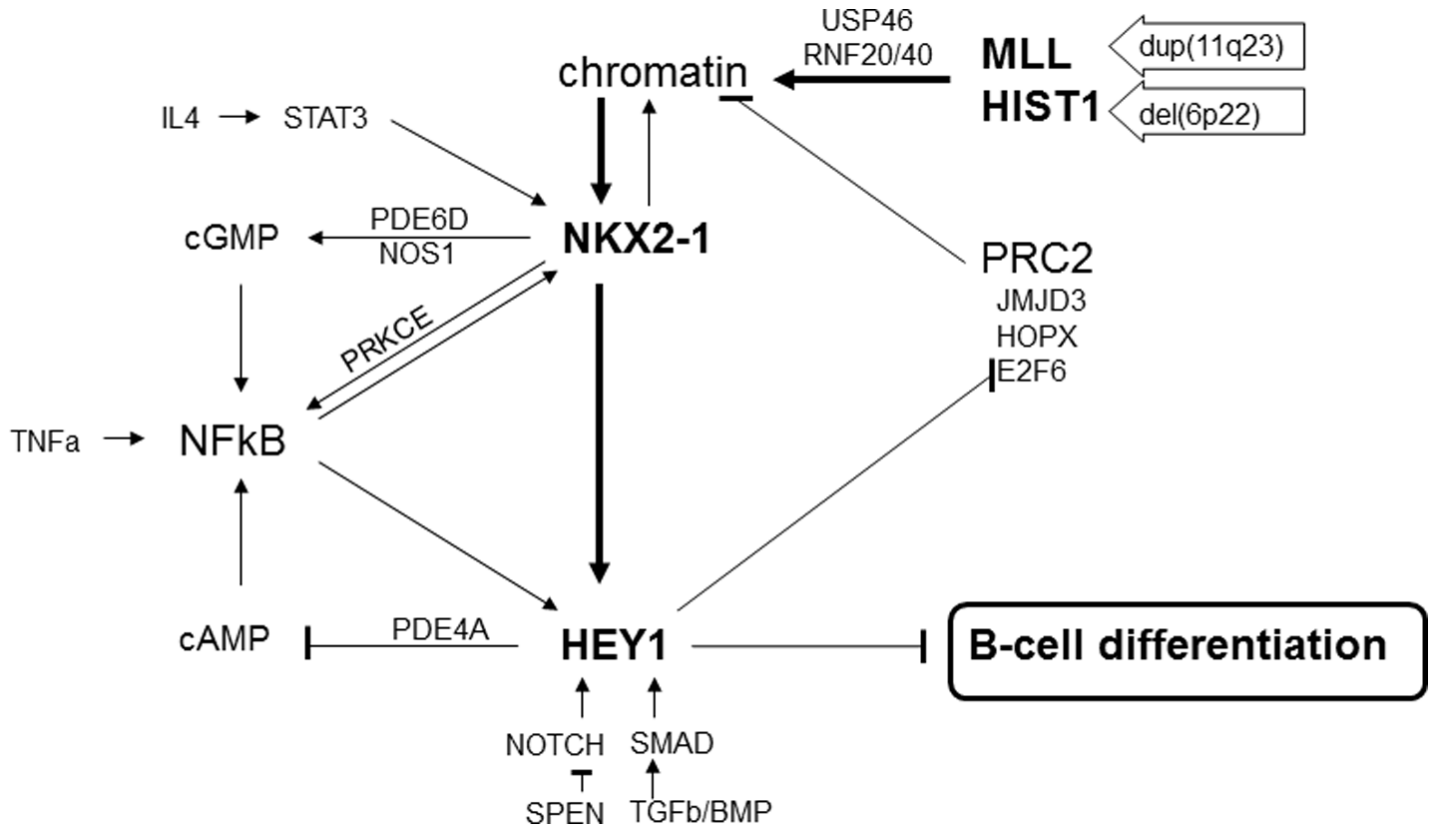
Oncogenic network of NKX2-1. The figure summarizes the regulations of the genes described in this study, highlighting a central position of NKX2-1. Chromosomal aberrations activate expression of MLL (11q23) and histones including H2B (6p22). MLL together with H2Bub1 generate an activatory chromatin structure at NKX2-1. This structure is reinforced by reduced expression of USP46 and E2F6 and elevated expression of RNF20/40, JMJD3 and HOPX, and mediates activation of NKX2-1. NKX2-1 in turn activates directly expression of HEY1 which performs inhibition of B-cell differentiation. Both, NKX2-1 and HEY1 contribute to the activatory chromatin structure by regulating RNF40 and E2F6, respectively. IL4/STAT3-signaling enhances expression of NKX2-1. TNFa, cGMP, cAMP and PRKCE support NFkB which activates both NKX2-1 and HEY1. Reduced expression of PDE6D and enhanced expression of NOS1 contribute to elevated cGMP. NOS1 and PRKCE are activated by NKX2-1. HEY1 mediates reduced expression of PDE4A resulting in elevated cAMP levels. Finally, NOTCH-signaling and TGFb-signaling (via SMAD) activate HEY1 expression. SMAD and NKX2-1 interact and coactivate HEY1 transcription.

Our data plot the emergence of an aberrant gene regulatory network with NKL homeobox gene NKX2-1 occupying a central role (**Fig. 6**). It comprises several network modules with feed-back motifs. The functional data indicate that these modules contribute to an enhancement and stabilization of NKX2-1 expression. In SU-DHL-5 deregulated chromatin may represent the initial step in NKX2-1 activation and subsequent cell transformation. According to such a model, chromosomal aberrations enhancing MLL and histones poised chromatin at the NKX2-1 locus for activation which subsequently regulates HEY1. This combination of modified chromatin and ectopic expression of transcriptional regulators represents an alternative mechanism of aberrant NKL homeobox gene activation in lymphoid malignancy. In T-ALL deregulation of select NKL family genes is typically effected by chromosomal alterations which juxtapose enhancer elements cognate to T-cell receptor genes or BCL11B [12]. In the case of NKX3-1, however, activation in T-ALL is controlled by particular deregulated hematopoietic TFs (TAL1, LYL1, MSX2) where aberrant chromatin structures may also participate [13], [14].

Transdifferentiation or reprogramming of cells is practicable in several cell types including hematopoietic cells and may be effected by forced expression of particular TFs [55]. For example CEBPA and GATA1 drive the differentiation into macrophages and megakaryocytes, respectively [56], [57], and NKX2-1 together with PAX8 mediate differentiation of embryonic stem cells into thyroid cells [58]. However, aberrant or ectopic expression of oncogenic (cell-type specific) TFs does not result in transdifferentiation of the tumor cells. These TFs rather disturb the physiological process of terminal differentiation, resulting in developmental arrest at immature stages. Explanations for the preference for differentiation arrest instead of reprogramming may be the cellular context as described for the TF TAL1 in T-ALL which resides at different binding sites in normal and leukemic cells or the need of stage-specific coregulators in addition to master factors [59], [60]. It is also likely that transdifferentiation requires permissive chromatin states normally present in embryonic cells which may be partially recapitulated in adult cells by treatment with histone methyltransferase inhibitors [61]. Noteworthy in this context is that the expression level of NKX2-1 was about 8-fold higher in primary physiological tissues as compared to SU-DHL-5. This scale of difference has been recognized for deregulated NKX2-5 and NKX3-1 in T-ALL as well [14], suggesting oncogenic actions of ectopic NKL homeobox genes at low expression levels instead of driving differentiation at higher levels. This interrelation suggests that enhancement of ectopic oncogene expression (e.g. NKX2-1 in DLBCL) may result in transdifferentiation of the lymphoma cells into benign non-hematopoietic cells, representing a novel concept for cancer therapy.

Taken together, we have identified aberrant expression of NKL homeobox gene NKX2-1 in subsets of DLBCL which is mediated by particular factors including TFs, chromatin mediators, and signaling components. This result expands the oncogenic role of this homeobox gene family within the group of lymphoid malignancies. However, diagnostic and/or therapeutic potentials require additional examinations. Nevertheless, our data may also be of interest for analyses and assessment of NKX2-1 in lung and thyroid cancer.

## Supporting Information

Figure S1 **Copy number and SKY data of HEY1.** Copy number analysis by genomic profiling indicates absence of aberrations at the locus of HEY1 at 8q13 in SU-DHL-5. The insert shows an enlargement of chromosome 8 obtained by SKY karyotyping.
(TIF)

Figure S2 **Copy number and SKY data of HOPX.** Copy number analysis by genomic profiling indicates absence of aberrations at the locus of HOPX at 4q12 in SU-DHL-5. The insert shows an enlargement of chromosome 4 obtained by SKY karyotyping.
(TIF)

Figure S3 **Methylation data for HOPX.** The locus of HOPX contains a CpG-island (CpG 109) as shown at the UCSC genome browser (above). RQ-PCR analysis of SU-DHL-5 cells treated with histone deacetylase-inhibitor TSA and DNA-methyltransferase-inhibitor 5-Aza-2′-deoxycytidine (AZA) (right). Data obtained by sequence analysis of bisulfite-treated DNA of SU-DHL-5 and SU-DHL-4 show no significant difference, demonstrating absence of HOPX deregulation via demethylated DNA at CpG 109. Each lollipop represents a CpG; filled lollipops represent methylated CpGs.
(TIF)

Table S1 **Oligonucleotides used for PCR.**
(DOC)
