# Correction: Ectopic Expression of Homeobox Gene NKX2-1 in Diffuse Large B-Cell Lymphoma Is Mediated by Aberrant Chromatin Modifications

**DOI:** 10.1371/annotation/3989404d-132f-4f07-a39e-c060d9094611

**Published:** 2013-06-26

**Authors:** Stefan Nagel, Stefan Ehrentraut, Jürgen Tomasch, Hilmar Quentmeier, Corinna Meyer, Maren Kaufmann, Hans G. Drexler, Roderick A. F. MacLeod

The label and legend for Figure 4 are incorrect. 

The correct label and legend are as follows: 

Figure 4: Chromatin modifiers. (A) RQ-PCR analysis of USP46 in DLBCL cell lines (left), siRNA-treated SU-DHL-5 cells (middle), and in SU-DHL-4 cells treated for overexpression of HEY1 (right). (B) RQ-PCR analysis of RNF20 (left) and RNF40 (right) in DLBCL cell lines. The data show abundant expression in SU-DHL-5. (C) RQ-PCR analyses show downregulation of RNF20 and NKX2-1 after siRNA-mediated knockdown of RNF20 (left). RQ-PCR analysis show downregulation of RNF40 and NKX2-1 after siRNA-mediated RNF20 knockdown (middle). RQ-PCR analysis shows downregulation of RNF40 after 26 siRNA-mediated knockdown of NKX2-1 (right). (D) RQ-PCR analysis of JMJD3 in DLBCL cell lines (left) and in SU-DHL-5 cells treated for siRNA-mediated knockdown of NKX2-1 (right). (E) RQ-PCR analysis of EZH2 in DLBCL cell lines (left). RQ-PCR analysis of SU-DHL-5 cells treated with EZH2-inhibitor DZNep shows upregulation of NKX2-1 (middle) and of HEY1 (right). (F) RQ-PCR analysis of HOPX in SU-DHL-5 (expression level was set to 1) and in leukemia/lymphoma control cell lines. (G) RQ-PCR analysis shows downregulation of HOPX and NKX2-1 after siRNA-mediated knockdown of HOPX (left). RQ-PCR analysis shows downregulation of NKX2-1 but not of HOPX after siRNAmediated knockdown of NKX2-1 (right). (H) RQ-PCR analysis of E2F6 in DLBCL cell lines (left) and in SU-DHL-5 cells treated for siRNA-mediated knockdown of HEY1 (right).

