# Supplementary figures and images for: Ectopic Expression of Homeobox Gene NKX2-1 in Diffuse Large B-Cell Lymphoma Is Mediated by Aberrant Chromatin Modifications
Source: PLoS One. 2013 Apr 29;8(4):e61447. doi: 10.1371/journal.pone.0061447 (PMC3639244; doi:10.1371/journal.pone.0061447)

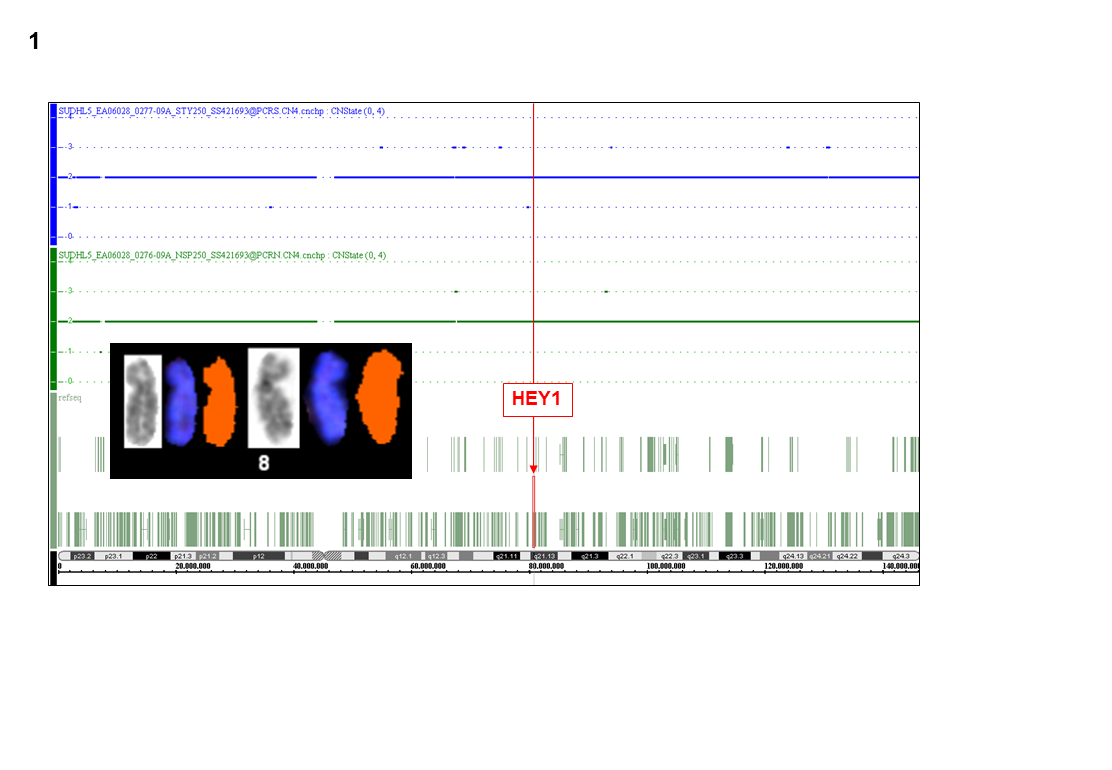

Supplement: Figure S1 — Copy number and SKY data of HEY1. Copy number analysis by genomic profiling indicates absence of aberrations at the locus of HEY1 at 8q13 in SU-DHL-5. The insert shows an enlargement of chromosome 8 obtained by SKY karyotyping. (TIF) [file pone.0061447.s001.tif]

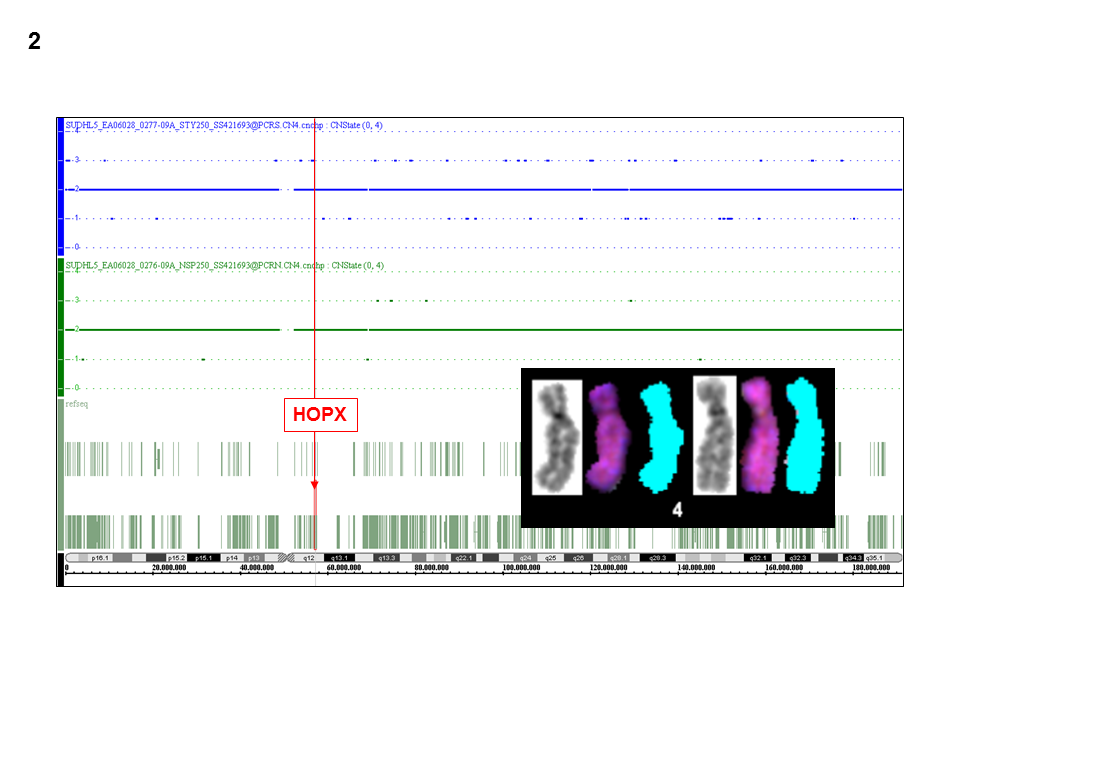

Supplement: Figure S2 — Copy number and SKY data of HOPX. Copy number analysis by genomic profiling indicates absence of aberrations at the locus of HOPX at 4q12 in SU-DHL-5. The insert shows an enlargement of chromosome 4 obtained by SKY karyotyping. (TIF) [file pone.0061447.s002.tif]

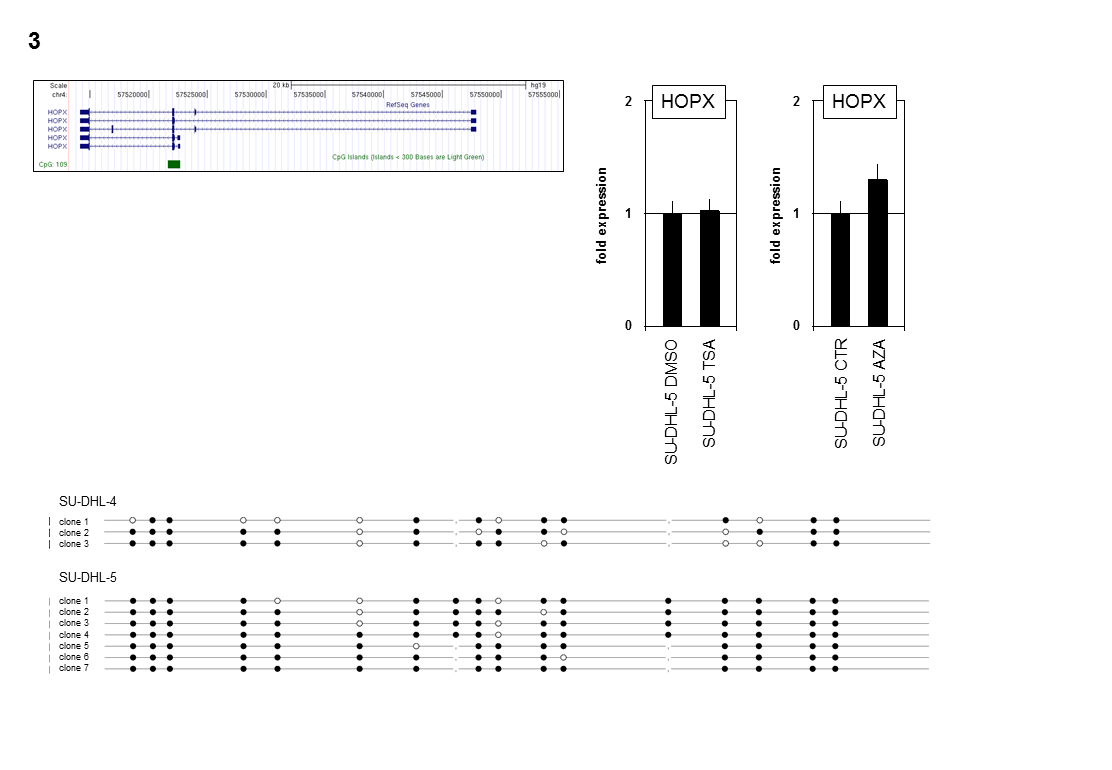

Supplement: Figure S3 — Methylation data for HOPX. The locus of HOPX contains a CpG-island (CpG 109) as shown at the UCSC genome browser (above). RQ-PCR analysis of SU-DHL-5 cells treated with histone deacetylase-inhibitor TSA and DNA-methyltransferase-inhibitor 5-Aza-2′-deoxycytidine (AZA) (right). Data obtained by sequence analysis of bisulfite-treated DNA of SU-DHL-5 and SU-DHL-4 show no significant difference, demonstrating absence of HOPX deregulation via demethylated DNA at CpG 109. Each lollipop represents a CpG; filled lollipops represent methylated CpGs. (TIF) [file pone.0061447.s003.tif]
