# Supplementary material for: Ectopic Expression of Homeobox Gene NKX2-1 in Diffuse Large B-Cell Lymphoma Is Mediated by Aberrant Chromatin Modifications
Source: PLoS One. 2013 Apr 29;8(4):e61447. doi: 10.1371/journal.pone.0061447 (PMC3639244; doi:10.1371/journal.pone.0061447)
Supplement: Table S1 — Oligonucleotides used for PCR. (DOC) [file pone.0061447.s004.doc]

**Table S1: Oligonucleotides used for PCR**

**Gene Acc.No Comment Forward (5´-3´) Reverse (5´-3´) .**

AF4 NM_005938 RT-PCR - CCTCAGGGTTCAGCATCCAC

AF6 NM_001207008 RT-PCR - CTCCGCTGACATGCACTTCATAG

AF9 NM_004529 RT-PCR - TACAGGCCTCTCCATTTCAG

AFX NM_005938 RT-PCR - CCTCAGGGTTCAGCATCCAC

ENL NM_005934 RT-PCR - CCTGACGAAGAGTCGTCCTC

HEY1 NM_012258 ChIP GGGTGATTCTTCAGGAGAGACG TTATGGATGCTCCAGTGCTTGG

Nested AAGGCAACACTTGCCCACTTCC GGAAGGCCTGTTCTGAGTTTGG

HOPX AF492681 Bisulfit TTAATGAATGTATTTAGGGGGA CCAAAAACCAACTAACCATAAA

MLL NM_005933 RT-PCR TCCAGAGCAGAGCAAACAGA -

NKX2-1 NM_003317 ChIP GGTTGCCTCGATGGCACTCG GGCTTGGTTTGTGCTAGGGATCC

Nested GCTCAGCCCGGCTTAGAAGC CAGGATGAGATCATGTGCCTGAG

NOS1 NM_000620 ChIP TGTAGCCTGGGAAGGGCAC TCTCTGAGCTCAGAGGAGCC

Nested GCCAGAGGCAGGTGAAGTCC CCGCCTTCCCGCTGCATTGG

PRKCE NM_005400 ChIP GGTTTGAGACCAACACTGTGAG TTCACCTGAAGTTCAGCTTTCC

Nested GGTGGCAGGAATGACTTGCCTC AGGATCCAGGCACCAAGTCC

TEL NM_001987 RT-PCR AGGCCAATTGACAGCAACAC TGCACATTATCCACGGATGG

**.**
